# Characterization on Polyester Fibrous Panels and Their Homogeneity Assessment

**DOI:** 10.3390/polym12092098

**Published:** 2020-09-15

**Authors:** Tao Yang, Ferina Saati, Jean-Philippe Groby, Xiaoman Xiong, Michal Petrů, Rajesh Mishra, Jiří Militký, Steffen Marburg

**Affiliations:** 1 Institute for Nanomaterials, Advanced Technologies and Innovation, Technical University of Liberec, 461 17 Liberec, Czech Republic; michal.petru@tul.cz; 2 Chair of Vibroacoustics of Vehicles and Machines, Department of Mechanical Engineering, Technical University of Munich, Boltzmannstrasse 15, 85748 Garching, Germany; ferina.saati@tum.de (F.S.); steffen.marburg@tum.de (S.M.); 3 Laboratoire d’Acoustique de l’Université du Mans, LAUM-UMR CNRS 6613, Le Mans Université, Avenue Olivier Messiaen, 72085 Le Mans CEDEX 9, France; Jean-Philippe.Groby@univ-lemans.fr; 4 Department of Material Engineering, Faculty of Textile Engineering, Technical University of Liberec, 46117 Liberec, Czech Republic; xiaoman.xiong@tul.cz (X.X.); rajesh.mishra@tul.cz (R.M.); Jiri.Militky@tul.cz (J.M.)

**Keywords:** Bayesian reconstruction, homogeneity, porous materials, polyester fibrous materials

## Abstract

Nowadays, fibrous polyester materials are becoming one of the most important alternatives for controlling reverberation time by absorbing unwanted sound energy in the automobile and construction fields. Thus, it is worthy and meaningful to characterize their acoustic behavior. To do so, non-acoustic parameters, such as tortuosity, viscous and thermal characteristic lengths and thermal permeability, must be determined. Representative panels of polyester fibrous material manufactured by perpendicular laying technology are thus tested via the Bayesian reconstruction procedure. The estimated porosity and airflow resistivity are found in good agreement with those tested via direct measurements. In addition, the homogeneity of polyester fibrous panels was characterized by investigating the mean relative differences of inferred non-acoustic parameters from the direct and reverse orientation measurements. Some parameters, such as tortuosity, porosity and airflow resistivity, exhibit very low relative differences. It is found that most of the panels can be assumed homogeneous along with the panel thickness, the slight inhomogeneity mostly affecting the thermal characteristic length.

## 1. Introduction

It has been demonstrated that polyester fibrous material is a good alternative to conventional sound absorbing material [1]. In textile industry, polyester fiber assemblies are in the form of woven, knitted and nonwoven structures. The woven structure fibrous material is made by using two or more sets of yarn interlaced to each other. Woven structure is generally more durable in comparison with knitted and nonwoven structures. However, woven structure is not widely used in noise treatment at low frequency range due to its relatively small thickness. The sound absorption of polyester woven structure determined by impedance tube and reverberant field method has been reported [2]. The single layer woven structure with a small thickness (i.e., 2.16–2.41 mm) exhibits poor sound absorption at low frequency range. Knitted structures are made of interlocking loops by using one or more yarns. Spacer fabrics, a special type of knitted structure, attracted great attention for sound absorption because of their thick structure possibility and designable appearances [3,4].

Unlike woven and knitted structures, nonwoven structure fibrous material has more advantages as a sound absorbing material, such as high porosity, economical price, light weight, recyclability, good elasticity and a large thickness range. Thus, nonwoven structure is more widely used for different noise reduction requirements. Nonwoven structure is produced in three stages: web formation, web bonding and finishing. Drylaid (i.e., carded, airlaid), wet-laid, spunmelt are the main technologies for web formation. Web bonding can be achieved through mechanical, thermal and chemical methods. The finishing aims to improve the outward appearance and the quality of the fibrous structure. Finishing methods are various according to different required specific properties. Nonwovens have an important role in sound absorption within the automotive, construction and a variety of industrial uses [5]. Nonwoven structure and its combination with other materials (such as woven structure, polyurethane foam, polypropylene foam, etc.) are used in seating area, headliners, side panels, carpets, trunks, bonnet liners for interior vehicle noise control [6]. Nonwoven structure material coupled with a hard wall can significantly reduce noise transmission and reduce the reverberation by improving the sound absorption [7,8].

The polyester nonwoven structure used in noise reduction is normally in the form of panel. The acoustic properties of polyester nonwoven panels have been well studied [9,10,11]. Kino et al. investigated the effect of various cross-sectional shapes polyester nonwoven structure on the acoustic and non-acoustic properties [10]. They concluded that cross-sectional shape has a slight effect on sound absorption, while there is a significant effect on the airflow resistivity, the thermal and viscous characteristic lengths. The accuracy of several prediction models for polyester materials was investigated by Garai and Pompoli, they recommended a new model to predict the acoustic characteristics of polyester fibrous materials [12].

Investigation of the homogeneity of an acoustic absorber can facilitate optimization of acoustic properties in practical applications. For instance, an absorber which is inhomogeneous in thickness direction will usually exhibit different acoustic performance at direct and reverse orientations. However, very few research studied characterization of the homogeneity of porous materials. In one paper, researchers applied X-ray computerized tomography (CT) to characterize the homogeneity of prebaked anode by analyzing the distribution of coke, pitch and porosity throughout the anodes as well as the variations in binder matrix thickness [13].

High-loft nonwoven panel is known to have useful acoustic properties [14]. Although some studies related to the acoustic properties of this material can be found in the literature [14,15], there is a lack of research on recovered non-acoustic parameters via inverse method for this type of material. Moreover, there are only a few publications focusing on characterization of the homogeneity of nonwoven panels. This paper presents an investigation of estimating non-acoustic parameters of polyester high-loft nonwoven panel via Bayesian reconstruction procedure. In addition, the homogeneity of polyester panels has been numerically assessed by comparing the results from panels’ direct and reverse orientations.

## 2. Experiment

### 2.1. Materials

**Figure 1:**
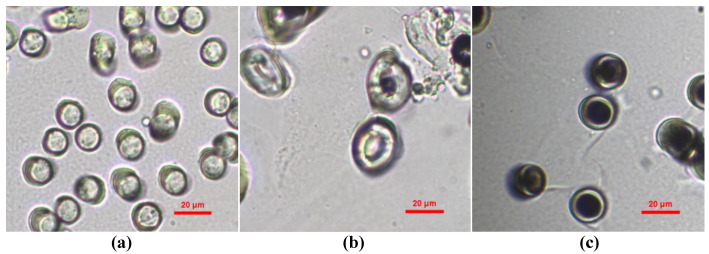
Cross-sectional images of fibers. (**a**) Staple fiber; (**b**) hollow fiber; (**c**) bicomponent fiber [9].

The polyester fibrous panels (Technical University of Liberec, Liberec, Czech Republic) are composed of 45 wt.% staple polyester, 30 wt.% hollow polyester and 25 wt.% bicomponent polyester. Low-melting polyester fiber consists of the sheath part of bicomponent fiber which is used to thermally bond the fiber structure. The cross-sectional images of three types of polyester fibers are shown in Figure 1. A microscope was used to measure fiber diameter, and fifty fibers were measured for each type of fiber to obtain an accurate average value. The mean diameters of the staple, hollow and bicomponent fibers are 13.19, 24.45 and 17.94 µm, respectively.

**Figure 2:**
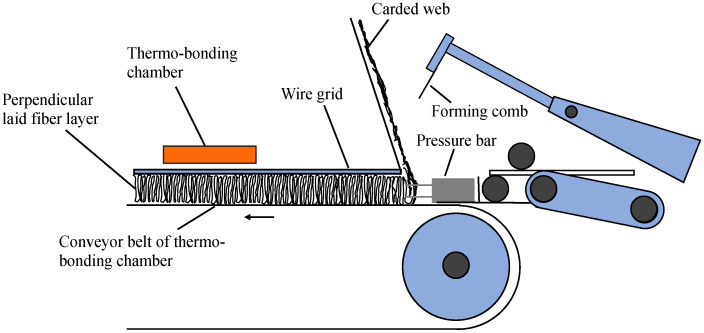
Sketch of perpendicular laying technology [17].

**Figure 3:**
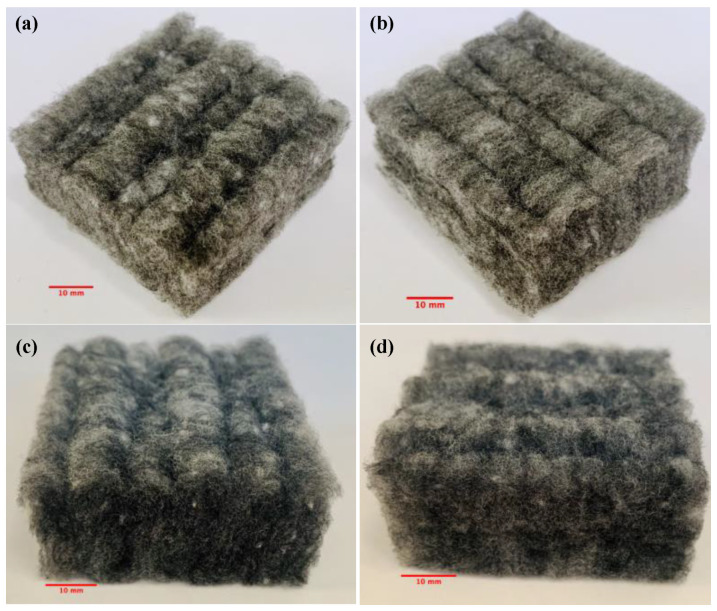
Example of manufactured polyester fibrous panel. Images are taken from different directions: (**a**,**b**) top view, and (**c**,**d**) side view.

Polyester fibrous panel samples were produced by perpendicular laying technology which consists of carded and thermal bonding procedures (see Figure 2) [16,17]. The carded web, consisting of a proportion of bicomponent polyester fibers in the blend, is fed onto the conveyor belt. The reciprocating forming comb and pressure bar are two main working elements used to create vertical folds. The forming comb strokes the lower part of the carded web and pushes the carded web to form a vertical fold. The reciprocating pressure bar moves the folded web along with the wire guide and the conveyor belt to the batt layer. With the movement of the pressure bar, the needles placed on the pressure bar penetrate the folded web and strengthens the folds, which improves the vertical orientation of the fibers in the nonwoven structure. The web is subsequently stabilized by melting the bonding fibers present in the fiber blend when it passes through the through-air bonding chamber. Thereafter, the nonwoven fabric is cooled. Panel thickness is controllable by setting the distance between the grid and conveyor as well as the dimension of the pressure bar. Panel density is adjusted via the velocity of the conveyor belt. By selecting proper fiber blend and adjusting the lapping device, various end products providing high absorption and insulation performance to meet a variety of applications could be achieved. An example of so manufactured polyester fibrous panel is illustrated in Figure 3.

### 2.2. Direct Characterization

Some non-acoustic parameters, such as thickness, porosity and airflow resistivity, can be easily determined according to ASTM D1777-96, ASTM C830-00 and ISO 9053–1, respectively [18,19,20]. The micro-CT (micro computed tomography), SEM (scanning electron microscope) and length weighted methods can be applied to determine fiber mean diameter [21,22]. The tomographic reconstruction method used to measure tortuosity of porous materials has been reported in 2009 [23].

Fiber diameter, thickness, porosity and airflow resistivity were directly measured in this study. The characteristics of the polyester fibrous panel specimens are listed in Table 1. An Alambeta device (SENSORA, Liberec, Czech Republic) was used to determine the panel thicknesses. Sample porosities were determined according to ASTM C830-00 [18]. In this standard, the porosity was determined as $\phi =1\;-\;\rho /\rho_{f}$, where ρ is the fabric bulk density and $\rho_{f}$ is the fiber density which is 1141.82 kg/m^3^. The density of three types of fibers was measured by liquid pycnometry technique [24]. Closed pores have less or no effect on airflow resistivity and sound absorption compared to open pores [25]. As a consequence, the voids in hollow fibers were not included in the analysis.

The 100 mm diameter circular specimens were punched by an Elektronische Stanzmaschine Type 208 machine for the standard airflow resistivity test. The airflow resistivity of samples was measured on AFD300 AcoustiFlow device (Gesellschaft für Akustikforschung Dresden mbH, Dresden, Germany) according to ISO 9053:1991 [20]. Ten samples were measured for each polyester nonwoven panel. Normally, the decrease of porosity results in the increase of airflow resistivity for the samples made by the same fiber content and manufacturing technology. While the airflow resistivity exhibits a drop in Samples 7–9, the phenomenon can be attributed to the slightly different manufacturing technology for these three samples compared to other samples.

### 2.3. Acoustic Characterization

The acoustic properties of the materials can be directly evaluated via reverberant chamber measurements, steady-state measurements and impedance tube measurements. Moreover, acoustic methods can be used to characterize the morphologic characterizations of reticulated foams, fibrous materials, granular materials and sandy soils [26].

#### 2.3.1. Impedance Tube Measurement

A four-microphone impedance tube was applied to carry out the measurements to recover the reflection *R* and transmission *T* coefficients. Then, the dynamic density ${\tilde{\rho }}_{eq}$ and dynamic bulk modulus ${\tilde{K}}_{eq}$ can be easily recovered [27,28,29].

**Figure 4:**
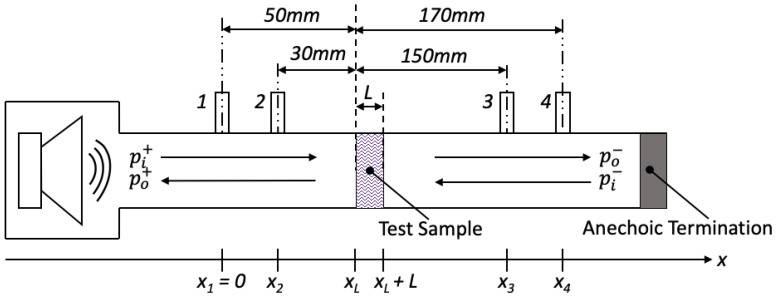
Four-microphone impedance tube configurations.

Figure 4 illustrates the 30 mm impedance tube used for this study. It consists of two 1/4 in. G.R.A.S. microphones flush mounted on both sides of the test sample and the tube ends with an anechoic termination. The distances between microphone positions and the sample front surface are 50, 30, 150 and 170 mm, respectively. The excitation signal was a logarithmic swept sine, over the frequency range of 800–5500 Hz. To achieve accurate results, the microphones were calibrated, and phase matched with each other [27].

#### 2.3.2. The Johnson-Champoux-Allard-Lafarge Model

In the Johnson-Champoux-Allard-Lafarge (JCAL) model, the equivalent dynamic density is associated with the viscous losses and the equivalent dynamic bulk modulus with the thermal losses [27]. The dynamic density of porous media was first proposed by Johnson et al. in 1987 [30]. Champoux, Allard and Lafarge et al. then modified the dynamic bulk modulus of porous media [31,32].

If saturating fluid is air, the JCAL model assumes the porous media are rigid and motionless at the frequency over the phase decoupling frequency. In the model, the equivalent dynamic density is described as:

(1) $${\tilde{\rho }}_{eq}=\frac{\rho_{0}}{\phi }\tilde{\alpha }\left(\omega \right)$$

where $\tilde{\alpha }\left(\omega \right)$ is the dynamic tortuosity, given by:

(2) $$\tilde{\alpha }\left(\omega \right)=\alpha_{\infty }+\frac{jv}{\omega }\frac{\phi }{k_{0}}\sqrt{1\;-\;\frac{j\omega }{v}{\left(\frac{2\alpha_{\infty }k_{0}}{\phi \Lambda }\right)}^{2}}$$

where $\rho_{0}$ is the density of the saturating fluid, ϕ is the open porosity, $\alpha_{\infty }$ is the dynamic tortuosity, j is the complex number, $v=\eta /\rho_{0}$ is the kinematic viscosity, where η is the dynamic viscosity, ω=2πf is the angular frequency, $k_{0}=\eta /\sigma$ is the static viscous permeability, where σ is the airflow resistivity, and Λ is the viscous characteristic length [30].

The dynamic bulk modulus is described as:

(3) $${\tilde{K}}_{eq}=\frac{\gamma P_{0}}{\phi }{\left(\gamma \;-\;\frac{\gamma \;-\;1}{{\tilde{\alpha }}^{\prime }\left(\omega \right)}\right)}^{\;-\;1}$$

where ${\tilde{\alpha }}^{\prime }\left(\omega \right)$ is the thermal tortuosity, given by

(4) $${\tilde{\alpha }}^{\prime }\left(\omega \right)=1+\frac{jv^{\prime }}{\omega }\frac{\phi }{k_{0}^{\prime }}\sqrt{1\;-\;\frac{j\omega }{v^{\prime }}{\left(\frac{2k_{0}^{\prime }}{\phi \Lambda \prime }\right)}^{2}}$$

where $v^{\prime }=\frac{v}{Pr}$, where Pr is the Prandtl number, $k_{0}^{\prime }$ is the static thermal permeability, $\Lambda^{\prime }$ is the thermal characteristic length [31,32].

#### 2.3.3. The Inversion Procedure

Inverse characterization methods are becoming more popular since they are able to simultaneously reckon several parameters [27]. The comparison between inferred porosity, airflow resistivity and characteristic lengths of porous materials using a commercial inversion software (i.e., FOAM-X) and those obtained from ultrasonic measurements, Bies-Allard and Kino-Allard models was studied in 2012 [33]. The deterministic inverse methods normally fit the measurements and predictions through models, then find the parameter that best describes the material. The Bayesian approach was applied to carry out the inversion procedure in this paper. Bayesian approach can inversely determine some physical parameters of porous materials from the impedance tube measurement [34]. The unknown parameters are assumed as random variables distributed according to a probability distribution in the Bayesian approach. This distribution is based on the existing knowledge or experience about the parameter values. However, for many of the non-acoustic parameters, it is possible to specify lower and upper bounds. Therefore, the prior parameter distribution can be defined. Posterior to collecting data, the parameter distribution is given by Bayes’ theorem. The posterior parameter distribution depends both on the prior distribution and on the measurements. Thus, this distribution contains all the available information about the parameters. By using the optimization method, good estimation of parameters can be obtained. One optimization technique called Markov chain Monte Carlo (MCMC) has been well applied to solve the problem of non-acoustic parameters estimation. Not only the probability density function (pdf) of each parameter, but also the joint probability density functions of the parameters can be retrieved according to the Bayesian approach in conjunction with MCMC [35].

### 2.4. Homogeneity Assessment

A homogeneous material has the same properties everywhere, i.e., uniform without irregularities. Some macroscopic pore structure parameters (e.g., permeability or porosity) can be used to verify the homogeneity of porous media [36]. However, homogeneity strongly depends on the selected sample size. At the initial increase of sample size, those parameters vary with the sample size and exhibit random fluctuations. As the sample size increases, the amplitude of the fluctuations gradually diminishes. The values of the pore structure parameters remain stable once a certain sample size is reached. Dullien [36] stated that when the structure parameters of a porous material maintain close values to the increasing sample size, the media is said to be macroscopically, or statistically, homogeneous.

As stated above, researchers characterized the homogeneity of prebaked anodes by analyzing the distribution of pores and different types of particles based on X-ray computerized tomography and image analysis [13]. However, the first step, i.e., computerized tomography on porous material, is a time-consuming work. For instance, one cubic sample with 3 mm × 4 mm × 10 mm cube lengths takes 6 to 8 h to accomplish the tomography. By contrast, the acoustic method is much more efficient. The inferred non-acoustic parameters (e.g., porosity, tortuosity, airflow resistivity) can represent the geometric structure in the polyester panels. Moreover, homogeneity in through-plane orientation is more important than in-plane orientation since sound waves mainly propagate from surface to inner structure. Thus, the homogeneity at thickness direction of polyester panels will be analyzed by comparing the inferred non-acoustic parameters.

## 3. Results

**Figure 5:**
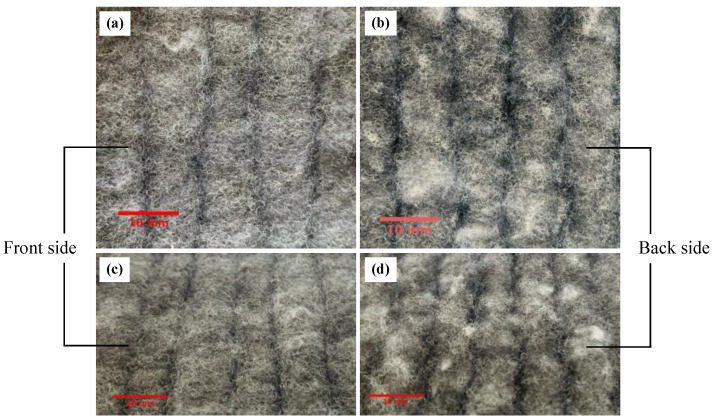
The surface texture of polyester fibrous panel sample: (**a**,**c**) front side, and (**b**,**d**) back side.

In order to figure out the homogeneity of polyester panels, the measurements from both direct and reverse orientations of samples have been carried out. The surface texture of front and back sides is shown in Figure 5.

In order to prepare specimens with proper radius, specimens are carefully cut by scissors. A transparent plastic tube having the same radius as the impedance tube was adapted to ensure the specimens fitting exactly into the tube’s cross-section. The samples are cautiously mounted in the connecting tube in the middle of four microphones before testing. The surface of each specimen is at the same level to the edge of the connecting tube. When changing the measurement direction, samples are rotated to another direction to avoid displacement. The front surface orientated to the speaker was referred to as direct orientation. The recovered parameters are listed in Table 2. Since airflow resistivity was used more often compared to static viscous permeability and these two parameters can be easily converted through the formula $k_{0}=\eta /\sigma$, only the airflow resistivity is presented in Table 2. In addition, the values of the recovered porosity (ϕ), tortuosity ($\alpha_{\infty }$), viscous characteristic length (Λ), thermal characteristic length ($\Lambda^{\prime }$) and static thermal permeability ($k_{0}^{\prime }$) are presented. The standard deviations for each value are reported in brackets. The tortuosity of common fibrous absorbent ranges from 1 to 1.06 [37]. As porosity approaches the value of 1, tortuosity reduces to the minimal value of 1 [38]. Some existing empirical formula between porosity and tortuosity, $\alpha_{\infty }=1/\sqrt{\phi }$ and $\alpha_{\infty }=1+\left(1\;-\;\phi \right)/2\phi$, can also explain this correlation [39,40]. The reconstructed tortuosity for all of the samples is 1 with low standard deviation as shown in Table 2. Although the recovered porosities seemingly display big differences compared to the directly measured values, the maximum relative error of inferred porosity is less than 4%. It was considered that the results with an error less than 10% are accurate enough for inverse analysis, as the value of porosity for a porous material can vary due to several uncertainties during measurements. Thus, it can be concluded that the inferred porosity is reasonable.

**Figure 6:**
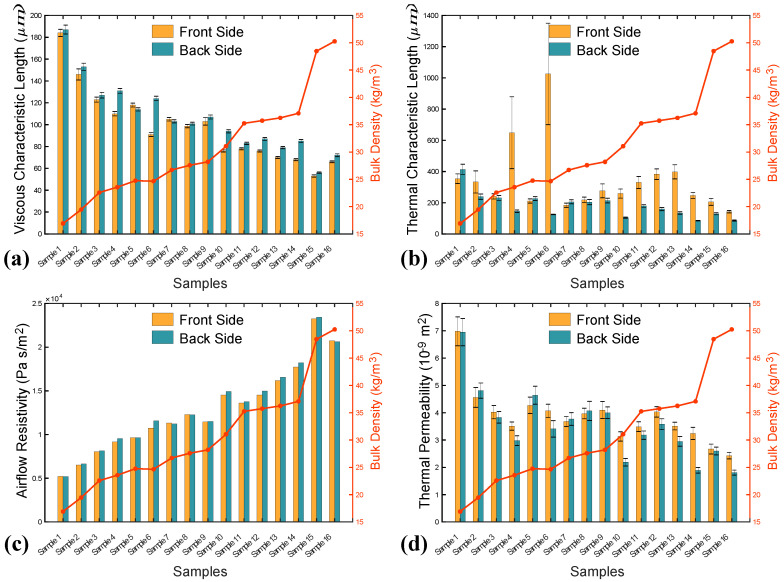
Inferred non-acoustic parameters from direct and reverse orientations: (**a**) viscous characteristic length Λ, (**b**) thermal characteristic length $\Lambda^{\prime }$, (**c**) airflow resistivity σ, and (**d**) thermal permeability $k_{0}^{\prime }$.

Figure 6 presents the comparison of some recovered non-acoustic parameters between two orientations. It can be easily seen that, with an increase of density the viscous characteristic length decreases, while the airflow resistivity increases. However, no clear trend can be found between thermal characteristic length, thermal permeability and density. Moreover, the difference on viscous characteristic length, airflow resistivity and thermal permeability are relatively small. It is obviously found that the inferred thermal characteristic length yields a significant difference on the two sides, especially for Samples 4 and 6 with densities of 23.54 kg/m^3^ and 24.54 kg/m^3^. In addition, the standard deviation is extremely high. This phenomenon can be attributed to the following reasons: interface difference on the two sides, complication on determination of thermal characteristic length and small frequency range or large measurement uncertainty [27]. The front side of samples is more even and continuous, while the back side is rough and uneven (see Figure 5). In addition, slight inhomogeneity can significantly affect thermal characteristic length. Estimation of the thermal characteristic length based on impedance measurement is very sensitive to boundary conditions. If the sample radius does not properly fit the impedance tube’s radius, the sample can be compressed, or air leakage existed between the sample and the tube. Consequently, erroneous values will occur because of the modified sample microstructure or the influence of air leakage on the inversion procedure.

**Figure 7:**
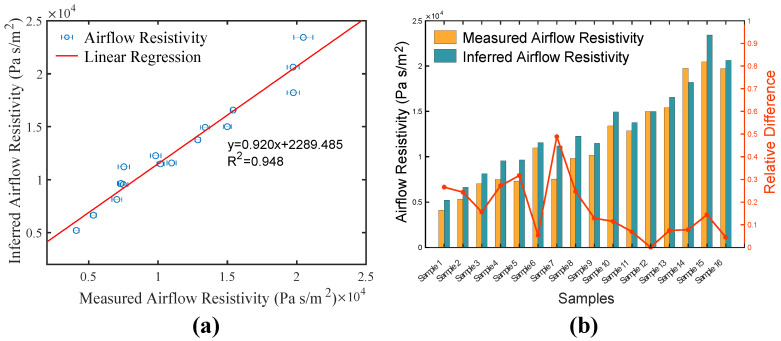
Comparison between measured and inferred airflow resistivity: (**a**) correlation between the measured and inferred values, and (**b**) their relative difference.

Since the values of inferred thermal characteristic length on the direct orientation are not reliable, the inferred airflow resistivity on the reverse direction was chosen to compare with the measured value in Figure 7. The correlation between the directly measured and recovered values is presented. It can be seen that the regression line has slope value close to 1 and the coefficient of determination is over 0.94. It can be further demonstrated that the recovering method can accurately estimate airflow resistivity of polyester fibrous panel. The relative difference was defined as $\delta =\left|\sigma_{meas}-\sigma_{infer}\right|/\sigma_{meas}$, where $\sigma_{meas}$ is the measured airflow resistivity and $\sigma_{infer}$ is the inferred value. The relative difference exhibits the biggest value for Sample 7 with a value of 0.489. This phenomenon can be explained by the small change that exists in manufacturing technology, as was stated above. The most accurate inversion of airflow resistivity occurs on Sample 12 with the smallest δ which is 0.001. As seen in Table 1, from Sample 1 to Sample 16 the density of the panels increases from 16.93 to 45.56 kg/m^3^. It can be found that δ is relatively lower when the samples are denser. For instance, Samples 9–16 have lower relative difference (e.g., <0.2). Meanwhile, among the low density panels (Samples 1–8) only two samples reach this relative difference level. It can be concluded that the inversion method of airflow resistivity is more accurate for denser polyester panel materials.

To compare the inferred non-acoustic parameters from direct and reverse orientations, the mean relative difference was calculated according to the following equation:

(5) $$\delta =\frac{1}{N}\overset{N}{\underset{n=1}{\sum }}\delta_{n}=\frac{1}{N}\sum_{n=1}^{N}\frac{\left|x_{f,n}-x_{b,n}\right|}{x_{f,n}}$$

where δ is the mean relative difference, $x_{f}$ and $x_{b}$ are, respectively the inferred non-acoustic parameters from direct and reverse orientations, and *N* is the total number of studied material specimens (*N* = 16).

**Figure 8:**
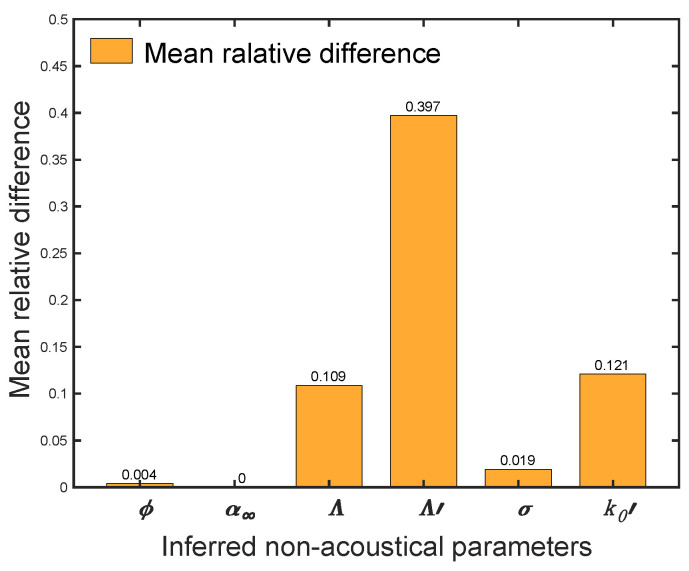
Mean relative differences of inferred non-acoustic parameters.

The mean relative differences of inferred porosity (ϕ), tortuosity ($\alpha_{\infty }$), viscous characteristic length (Λ), thermal characteristic length ($\Lambda^{\prime }$), airflow resistivity (σ) and thermal permeability ($k_{0}^{\prime }$) are presented in Figure 8. The values of mean relative differences are demonstrated on the top of each bar. It was assumed that the material is homogeneous when the physical parameters having variances less than 0.05 [41]. The mean relative differences of tortuosity, porosity and airflow resistivity are much smaller than the critical value. The differences of viscous characteristic length and thermal permeability are 0.109 and 0.121, respectively. However, the inferred thermal characteristic length exhibits the highest mean relative difference with the value of 0.397. Due to the sensitivity of estimating thermal characteristic length, its inferred values are not recommended for assessing materials homogeneity. Furthermore, tortuosity, porosity and airflow resistivity can well represent the pore size, fiber size and their distributions. Thus, the polyester fibrous sample panel is nearly homogeneous or slightly inhomogeneous at thickness direction. Moreover, the acoustic method can be an alternative approach to characterize the homogeneity of porous material. It can be used not only in the thickness direction, but also in arbitrary directions. Nevertheless, the size of the sample and the suitability of the JCAL model for the porous material of interest should be considered.

## 4. Conclusions

This work applied a Bayesian approach on polyester fibrous materials to inversely estimate some non-acoustic parameters. Meanwhile, the homogeneity of polyester nonwoven panels was assessed by comparing the inferred non-acoustic parameters from direct and reverse orientations. The Johnson-Champoux-Allard-Lafarge model and Markov chain Monte Carlo optimization technique were chosen to implement the Bayesian approach. Polyester samples with density ranging from 16.93–45.56 kg/m^3^ were selected in this study. Measurements of reflection coefficient and transmission coefficient were carried out in a four-microphone impedance tube. The mean relative differences of inferred parameters between two orientations were used to analyze the homogeneity. The results indicated that the estimated porosity and tortuosity are reasonable. Moreover, other assessed parameters have generally lower contrast and standard deviation by looking at the qualities from two directions, while a sizable difference of thermal characteristic length was found. In addition, the increase in density decreases estimates of viscous characteristic length and increases estimates of airflow resistivity. While density does not show clear connections with thermal characteristic length and thermal permeability. Measured airflow resistivity and inferred values are very close. The inverse method can accurately estimate the non-acoustic parameters of denser polyester fibrous panel material (i.e., density > 28 kg/m^3^). Slight inhomogeneity could significantly affect the determination of thermal characteristic length. The mean relative differences of inferred tortuosity, porosity and airflow resistivity are, respectively 0, 0.004 and 0.019 which means the polyester material is nearly homogeneous. The applied acoustic method is an efficient way to characterize homogeneity of porous materials by comparing with computerized tomography technology. Hence, the proposed Bayesian inversion based on impedance tube measurement is valuable for the study on characterization of the homogeneity of porous material.

## Figures and Tables

**Table 1:** Characteristics of polyester materials.

Samples	Porosity (%)	Bulk Density (kg/m^3^)	Thickness (mm)	Airflow Resistivity (Pa·s/m²)
Sample 1	98.52	16.93	27.48	4108 ± 199
Sample 2	98.29	19.49	23.87	5357 ± 217
Sample 3	98.03	22.49	20.69	7029 ± 356
Sample 4	97.94	23.54	20.32	7498 ± 333
Sample 5	97.86	24.45	20.76	7319 ± 243
Sample 6	97.85	24.54	19.49	10,978 ± 329
Sample 7	97.66	26.71	19.00	7530 ± 408
Sample 8	97.59	27.54	18.43	9829 ± 376
Sample 9	97.58	27.61	16.85	10,181 ± 259
Sample 10	97.29	30.94	15.46	13,397 ± 329
Sample 11	96.94	34.95	13.31	12,868 ± 199
Sample 12	96.89	35.56	14.27	14,989 ± 285
Sample 13	96.86	35.87	14.15	15,414 ± 167
Sample 14	96.59	38.98	12.27	19,751 ± 442
Sample 15	96.09	44.60	10.43	20,474 ± 687
Sample 16	96.01	45.56	11.14	19,733 ± 688

**Table 2:** Inferred non-acoustic parameters of polyester panels.

Samples	Orientations	ϕ (%)	$\alpha_{\infty }$	Λ (μm)	$\Lambda^{\prime }$ (μm)	σ (Pa·s/m_2_)	$k_{0}^{\prime }$ (10^−9^ m_2_)
Sample 1	Direct	99.8 (0.19)	1(0.0017)	184 (3.2)	354(30.8)	5225	6.98 (0.53)
	Reverse	99.8 (0.18)	1(0.0017)	187 (4.0)	414(32.0)	5202	6.95 (0.50)
Sample 2	Direct	99.0 (0.86)	1 (0.0026)	146 (5.1)	333(71.0)	6527	4.56 (0.36)
	Reverse	99.8 (0.19)	1 (0.0018)	153 (3.3)	239 (16.9)	6638	4.81 (0.28)
Sample 3	Direct	99.6 (0.30)	1 (0.0027)	123 (2.3)	241 (16.7)	8052	4.02 (0.24)
	Reverse	99.6 (0.35)	1(0.0029)	127 (2.6)	231 (15.6)	8129	3.83 (0.21)
Sample 4	Direct	99.7 (0.27)	1 (0.0027)	110 (2.0)	649 (230.0)	9147	3.51 (0.15)
	Reverse	99.7 (0.18)	1 (0.0016)	131 (2.2)	146 (7.7)	9538	2.98 (0.18)
Sample 5	Direct	99.8 (0.18)	1 (0.0016)	118 (1.8)	211 (13.7)	9650	4.27 (0.30)
	Reverse	99.8 (0.17)	1(0.0012)	114 (1.5)	227 (12.2)	9641	4.64 (0.33)
Sample 6	Direct	98.9 (0.54)	1 (0.0035)	91 (1.8)	1025 (325.0)	10,754	4.07 (0.24)
	Reverse	100 (0.03)	1 (0.0012)	124 (2.0)	125 (2.2)	11,576	3.41 (0.30)
Sample 7	Direct	99.8 (0.13)	1 (0.0011)	105 (1.6)	184 (13.5)	11,313	3.68 (0.18)
	Reverse	99.8 (0.14)	1 (0.0014)	103 (1.4)	206 (12.9)	11,213	3.77 (0.23)
Sample 8	Direct	99.8 (0.18)	1 (0.0012)	98.7 (1.5)	219 (17.4)	12,283	3.97 (0.19)
	Reverse	99.8 (0.15)	1 (0.0013)	101 (1.3)	204 (16.3)	12,259	4.07 (0.35)
Sample 9	Direct	97.0 (0.95)	1 (0.0024)	103 (3.4)	276 (44.4)	11,434	4.10 (0.31)
	Reverse	99.8 (0.25)	1 (0.0022)	107 (1.8)	214 (15.3)	11,491	4.00 (0.21)
Sample 10	Direct	99.7 (0.24)	1 (0.0020)	76 (1.0)	259 (28.9)	14,528	3.13 (0.17)
	Reverse	99.8 (0.12)	1 (0.0011)	94 (1.4)	104 (4.0)	14,935	2.19 (0.14)
Sample 11	Direct	99.7 (0.23)	1 (0.0018)	78 (0.9)	330 (38.3)	13,613	3.49 (0.18)
	Reverse	99.8 (0.18)	1 (0.0012)	83 (1.0)	180 (8.9)	13,763	3.18 (0.15)
Sample 12	Direct	99.8 (0.13)	1 (0.0013)	76 (0.9)	383 (34)	14,535	4.03 (0.19)
	Reverse	99.8 (0.14)	1 (0.0018)	87 (1.3)	159 (9.6)	15,004	3.58 (0.20)
Sample 13	Direct	99.8 (0.24)	1 (0.0011)	70 (0.9)	398 (45)	16,167	3.51 (0.14)
	Reverse	99.8 (0.15)	1 (0.0020)	79 (1.0)	134 (7.6)	16,567	2.95 (1.77)
Sample 14	Direct	99.8 (0.16)	1 (0.0017)	68 (1.0)	246 (19.4)	17,734	3.24 (0.22)
	Reverse	99.9 (0.08)	1 (0.0014)	85 (1.4)	86 (1.6)	18,205	1.89 (0.95)
Sample 15	Direct	98.3 (0.76)	1 (0.0041)	53 (1.1)	206 (21.9)	23,241	2.67 (0.18)
	Reverse	99.8 (0.16)	1 (0.0016)	56 (0.81)	130 (6.9)	23,432	2.60 (0.14)
Sample 16	Direct	99.8 (0.14)	1 (0.0020)	66 (0.80)	143 (8.0)	20,712	2.43 (0.12)
	Reverse	99.8 (0.14)	1(0.0017)	72 (1.2)	86 (4.1)	20,619	1.81 (0.09)
